# A Beta Regression Approach to Modelling Country-Level Food Insecurity

**DOI:** 10.3390/foods14172997

**Published:** 2025-08-27

**Authors:** Anamaria Roxana Martin, Tabita Cornelia Adamov, Iuliana Merce, Ioan Brad, Marius-Ionuț Gordan, Tiberiu Iancu

**Affiliations:** 1 Doctoral School Engineering of Plant and Animal Resources, University of Life Sciences “King Mihai I” from Timisoara, Calea Aradului No. 119, 300645 Timisoara, Romania; anamaria.martin@usvt.ro; 2 Faculty of Management and Rural Tourism, University of Life Sciences “King Mihai I” from Timisoara, Calea Aradului No. 119, 300645 Timisoara, Romania; tabitaadamov@usvt.ro (T.C.A.); iulianamerce@usvt.ro (I.M.); ioanbrad@usvt.ro (I.B.); tiberiuiancu@usvt.ro (T.I.)

**Keywords:** food insecurity, food insecurity determinants, Sustainable Development Goals, Zero Hunger, beta regression

## Abstract

Food insecurity remains a persistent global challenge, despite significant advancements in agricultural production and technology. The main objective of this study is to identify and quantitatively assess some of the structural determinants influencing country-level food insecurity and provide an empirical background for policy-making aimed at achieving the Sustainable Development Goal of Zero Hunger (SDG 2). This study employs a beta regression model in order to study moderate or severe food insecurity across 153 countries, using a cross-sectional dataset that integrates economic, agricultural, political, and demographic independent variables. The analysis identifies low household per capita final consumption expenditure (β = −9 × 10^−5^, *p* < 0.001), high income inequality expressed as a high GINI coefficient (β = 0.047, *p* < 0.001), high long-term inflation (β = 0.0176, *p* = 0.003), and low economic globalization (β = −0.021, *p* = 0.001) as the most significant predictors of food insecurity. Agricultural variables such as land area (β = −1 × 10^−5^, *p* = 0.02) and productivity per hectare (β = −9 × 10^−5^, *p* = 0.09) showed limited but statistically significant inverse effects (lowering food insecurity), while factors like unemployment, political stability, and conflict were not significant in the model. The findings suggest that increased economic capacity, inequality reduction, inflation control, and global trade integration are critical pathways for reducing food insecurity. Future research could employ beta regression in time-series and panel analyses or spatial models like geographically weighted regression to capture geographic differences in food insecurity determinants.

## 1. Introduction

Food security was defined during the 1996 World Food Summit as the state in which every individual has “at all times, physical and economic access to sufficient, safe and nutritious food to meet their dietary needs and food preferences for an active and healthy life” [1,2]. Although agricultural technologies have sustained continuous development in recent years and the performance of agricultural production has increased substantially over the last century, food insecurity remains a significant issue worldwide [3]. Paradoxically, while the population of developed countries and part of those in developing countries are in the positive segment of food security and generate enormous amounts of waste from food waste, at the same time, the population of underdeveloped countries, as well as part of those in developing countries, suffers from malnutrition and undernutrition leading to health issues or death by starvation [4,5].

It has long since been argued that worldwide food production is enough to feed the entire population, but the fact remains that food production and distribution is not distributed evenly or fairly [6]. For this reason, in many parts of the world, hunger poses a danger to the respective human populations. Globally, enough food is produced to provide every man, woman, and child with more than 2300 kilocalories per day, which can be considered more than sufficient [7,8]. At the same time, if current trends are to hold, the prospects are not good at all, and humanity is not on track to meet the goals for the food security indicators by 2030 [9,10,11]. The most recent State of Food Security and Nutrition in the World (SOFI) report estimates that about 733 million people (1 in 11 globally) experienced undernourishment in 2023 [4]. Approximately 9 million deaths per year are attributed to hunger, averaging around 25,000 deaths each day, including thousands of children [12,13]. Food prices and overall inflation continue to deepen vulnerabilities in food security; World Bank data indicate that each 1% increase in global food prices pushes an additional 10 million people into extreme poverty, endangering food security for millions more [13,14].

Food insecurity can become a more pressing issue given current demographic trends. The United Nations World Population Prospects projects an increase in the global population from around 8 billion in 2025 to 10.2 billion by 2086 [15]. Such questions regarding the relation between the rate of population growth and the improvement of resource usage (and, in turn, of food supply efficiency) have long since been debated, dating back to Thomas Malthus’ works [16,17]. Malthus warned that population growth tends to outpace food production, leading to widespread scarcity and hardship unless kept in check by some natural or societal constraints. Although technological advancements in agriculture have so far prevented Malthus’s predictions from materializing, his concerns remain relevant, especially in regions where agricultural innovation lags behind demographic expansion [18]. Enhancements in food production efficiency, sustainable usage of natural resources, and the overall reduction and repurposing of food and agricultural waste are viable pathways to preventing a Malthusian crisis [19]. Urgent, effective, and efficient measures need to be taken to support disadvantaged communities, or else the scale of the disaster regarding hunger, malnutrition, and loss of livelihoods will be devastating [20,21].

Modern food-security theory can be traced to Amartya Sen’s entitlement approach, which shifted attention from aggregate food availability to people’s command over food through incomes, prices, and rights [22]. Based on this, FAO articulated four interdependent pillars: availability (is food physically present?), access/affordability (can households obtain it?), utilization (can nutrients be absorbed given diet/health?), and stability (are availability and access sustained over time?) [23]. This theoretical framework guides the variable selection process by providing a policy-endorsed, comparable, and operational schema that aligns with our outcome measure (SDG 2.1.2/FIES, primarily an access indicator), permits consistent cross-country measurement for 153 countries, and yields a parsimonious set of regressors that map directly to actionable policy levers (for example, trade globalization), thereby addressing a significant knowledge gap.

Given this context, the primary objective of this study is to investigate and identify some of the structural determinants of national-level food insecurity through quantitative modeling. Specifically, we apply beta regression techniques to evaluate how economic, agricultural and food-related, political, demographic, and disaster-based factors contribute to food insecurity globally. The relevance of this study lies in its comprehensive statistical approach, integrating multiple dimensions that have previously been considered in isolation, and its consideration of variables describing almost all countries.

The study’s novelty lies in employing beta regression modeling—a method not previously used in studies concerning food insecurity determinants at the national level, which is more suitable for ratio dependent variables, such as moderate or severe food insecurity, compared with Ordinary Least Squares regression [24]. Ultimately, our research provides a theoretical and empirical framework for policymakers aiming to reach the Sustainable Development Goal of Zero Hunger (SDG 2) through informed, targeted, and evidence-based interventions.

The structure of the paper can be described as follows:

1. Introduction, where basic background information is covered.

2. Theoretical and policy background, which outlines the conceptual, empirical, and policy background and motivates variables selection.

3. Materials and methods details data sources and data cleaning methods used, and the beta-regression specification.

4. Results reports descriptive statistics, regression coefficient and statistical significance results, and marginal effects.

5. In the Discussion section, we elaborate on interpretation, policy implications, limitations, and relations to prior studies.

6. The Conclusions section describes future research avenues and summarizes the findings of the paper.

## 2. Theoretical and Policy Background

### 2.1. Sustainable Development Goal 2 (Zero Hunger)

Sustainable Development Goal 2, as outlined by the United Nations, aims to “End hunger, achieve food security and improved nutrition and promote sustainable agriculture” [10,25]. Specifically, Target 2.1 of this goal states: “By 2030, end hunger and ensure access by all people, in particular the poor and people in vulnerable situations, including infants, to safe, nutritious and sufficient food all year round”. Progress towards this target is quantified through two indicators: 2.1.1. Prevalence of undernourishment and 2.1.2. Prevalence of moderate or severe food insecurity in the population, based on the Food Insecurity Experience Scale (FIES), respectively. Our research will focus on the latter.

Indicator 2.1.2 measures the prevalence of moderate or severe food insecurity in the population, as determined by the Food Insecurity Experience Scale (FIES) in the UN Sustainable Development Goals framework. This indicator measures the proportion of individuals in a specific country who experienced moderate or severe food insecurity during the specified reference period (annually). Moderate food insecurity is typically characterized by an inability to consistently consume healthy, balanced diets. In contrast, severe food insecurity typically indicates a reduction in food consumption, leading to more dangerous manifestations of undernutrition, including hunger [26,27].

It should be noted that the latest progress report for the 2.1. target, which analyzed data from 2023, outlines the fact that global hunger has risen since 2019, and that 1 in 11 people worldwide suffer from hunger [28].

An analysis of moderate or severe food insecurity aggregated at the regional level is presented as follows (Table 1):

At the global level, the prevalence of moderate or severe food insecurity increased from 21.7% in 2015 to 29.0% in 2022, a rise of 7.3% in absolute percentage and of 33.64% in relative terms. The period culminates in a broad-based peak in 2021–2022, consistent with overlapping global shocks that strained household purchasing power and food-system stability, which included a strained geopolitical context as well as the COVID-19 pandemic. While deterioration is widespread, regional trajectories differ meaningfully in both magnitude and timing, indicating a heterogeneous exposure and resilience across different contexts.

All African subregions experienced increases, but with marked variation in both levels and changes. Western Africa recorded the steepest deterioration globally (increasing by 21.0%), rising from 39.7% to 60.7%, and passing several inflection points in 2019–2021 that align with cumulative pressures on food security. Eastern Africa also registered a substantial increase (+6.9%), moving from an already high 58.5% to 65.4%, with a continuous rise through 2018 and renewed increases in 2019–2021. Middle Africa, for which consistent data are available from 2019, shows a high and rising profile (69.5% to 76.7%, +7.2%). Southern Africa exhibits a smaller but steady increase (+2.6 pp, 21.5% to 24.1%), suggesting persistent, although comparatively milder, pressures. Northern Africa food insecurity rose by 4.8% (28.6% to 33.4%). Taken together, the African pattern points to a generalized food security challenge, with Western Africa’s rapid deterioration and Eastern/Middle Africa’s persistently high levels standing out as priority areas.

Latin America and the Caribbean food insecurity increased by 6.2% overall (25.1% in 2015 to a peak of 33.4% in 2021, then moderating to 31.3% in 2022), indicating a pronounced but partially reversible deterioration. Within the region, the levels in South America rose by 9.5% (19.7% to a 2021 peak of 31.4%, then 29.2% in 2022), while Central America experienced a net increase of 0.4% over the period (with notable heterogeneous dynamics: decreases through 2017, increases to 2020, then renewed declines). This suggests that cyclical shocks translated into food-insecurity risk, but with varying persistence across subregions. In contrast, Northern America improved modestly (–0.9%), trending downward through 2020 before a slight uptick in 2021–2022; overall levels remained comparatively low (8–10%), consistent with higher economic development and social protection mechanisms buffering households against price spikes.

The development of Asian food insecurity is differentiated by subregion. Southern Asia shows a sharp increase (+13.7%, from 27.6% to 41.3%), with the highest values reached in 2021; this is among the largest regional deteriorations globally and signals a significant stress on affordability for large populations. Western Asia levels rose by 8.2% (30.7% to 38.9%), with a sustained increase through 2021 and a plateau thereafter. Central Asia levels increased by 8.8% (9.2% to 18.0%), also peaking in 2021 before easing slightly. South-eastern Asia exhibits a moderate net rise (+2.2%, 14.8% to 17.0%), characterized by early increases, a 2018 pullback, and resumed growth thereafter—an arc suggestive of partial resilience but lingering exposure to external shocks. By contrast, Eastern Asia displays relative stability (+0.2% net change, ranging 6–9%) with a 2018 peak followed by successive declines; this profile indicates that improvements in stability and purchasing power helped offset earlier pressures. Overall, Asia’s pattern reveals large deteriorations concentrated in the South and West, with Central Asia also adversely affected, while the East shows limited net change over the whole period.

Europe remains the region with the lowest prevalence of food insecurity, and three subregions improved over the period. Eastern Europe food insecurity levels declined by 0.6% (11.2% to 10.6%), with a sharper decline in the mid-period and stability thereafter. Northern Europe food insecurity fell by 0.4% (6.7% to 6.3%), following a multi-year decline that reversed slightly in 2021–2022. Southern Europe showed a decrease by 0.9% (7.4% to 6.5%), with a downward trend from 2018 onward. Western Europe food insecurity levels increased slightly (+0.4%, 5.2% to 5.6%), driven by a post-2020 rise from previously lower levels. Despite these small changes, Europe’s consistently low prevalence suggests that increased economic development mitigated the translation of price and income shocks into moderate or severe food insecurity.

Levels in Oceania rose by 2.8% (22.2% to 25.0%), with a steady increase through 2018, a plateau during 2019–2021, and a renewed rise in 2022.

Based on those facts, three broad observations emerge. First, the global increase is primarily an affordability phenomenon; the steepest deteriorations are concentrated in regions with lower household consumption capacity and higher vulnerability to sustained inflation, exchange-rate movements, and imported price shocks [30,31,32]. Second, many regions registered their highest values in 2021–2022, indicating that the confluence of global disruptions had lingering effects on household purchasing power and diet adequacy. Third, there is heterogeneous persistence of trends: some regions (e.g., parts of the Americas and Eastern Asia) exhibit partial reversals after peaks, while others (notably Western and Southern Asia and much of Africa) remain elevated, showing structural constraints that go beyond short-lived price spikes, such as baseline affordability/inequality and the high cost of healthy diets, persistent inflation, trade integration and logistics resilience that hasten domestic price realignment, and conflict and recurrent climate shocks that convert transitory disturbances into protracted crises [33,34,35,36,37].

### 2.2. Outline of Possible Factors Influencing Food Insecurity

To address the SDG 2 goal of eliminating hunger worldwide by 2030, it is crucial to identify and address the underlying factors that drive the prevalence of moderate and severe food insecurity. A comprehensive, evidence-based approach is critical for developing sustainable solutions that effectively reach the most vulnerable populations and ultimately lead to better policy outcomes.

Variables were selected to cover the theoretical framework of the four food-security pillars [23,38]. In our framework, availability is proxied by total agricultural land, agricultural value per hectare, the share of agricultural land, and externally, economic globalization, which broadens sourcing and input flows in the agricultural sectors, and cereal import dependency, which can either buffer or heighten exposure depending on diversification. Access is captured by per capita HFCE (purchasing power), the Gini index (distributional barriers), unemployment (income loss), and urbanization (better market access but greater reliance on purchased food). Urbanization is the only proxy for utilization, given that the dependent variable is itself an expression of utilization. Stability is represented by long-run inflation, food-price anomalies, political stability/violence, armed conflict, disaster damages, and again economic globalization as a diversification buffer.

Furthermore, variables were chosen for consistent coverage and comparability for the studied period across 153 countries, and parsimony under multicollinearity constraints (e.g., using the KOF economic globalization subindex instead of the global composite index to avoid overlap). This yielded an economic category of variables (consumption metrics, inequality, inflation, globalization, unemployment), a food and agriculture category (land, production per hectare, cereal import dependency, food price anomalies), governance/conflict, demography/urbanization, and disasters.

The variables considered are shown in Table 2.

The first variable type assessed is economic variables, as follows:

The Gini index, formulated by Corrado Gini in 1912, is the most widely used measure to assess income distribution and is also applied to approximate other distributions like consumption and wealth [39,40]. It is the most widely used measure of inequality in the world, employed by nearly all governments and international organizations to express income or wealth inequality within a nation or globally. It ranges from 0, implying perfect equality (when everyone has the same income or wealth), to 1, implying perfect inequality (when all the income or wealth is concentrated in one recipient or a group of recipients). The Gini coefficient is measured as the distance between the Lorenz curve, the actual cumulative income distribution, and the perfectly equal income distribution [41].

Inflation is an increase in the overall level of prices of goods and services usually bought by families. The most commonly recognized measure of inflation is the Consumer Price Index (CPI), which monitors fluctuations in the prices of the goods and services usually purchased by households [42]. We expect a positive relationship between inflation and food insecurity due to the decrease in purchasing power caused by inflationary pressures [43].

In our analysis, we assessed the impact of annual average long-term inflation (over the course of 9 years, from 2015 to 2023) [19]. We used an average inflation rate to account for the fact that inflation is a multiplicative, not additive, process (inflation rates are calculated based on prices reported from the previous year). We expect low yearly inflationary pressures to have a lesser impact on food insecurity compared to consistently high inflation rates.

The KOF Globalization Index, established by Dreher in 2006, measures globalization in the economic, social, and political dimensions for nearly all nations worldwide since 1970. It has emerged as the preeminent index of globalization in academic literature [44,45,46]. The updated KOF Globalization Index adds a separation between de facto and de jure indicators of globalization. While de facto measurements of globalization include “variables that describe flows and activities, de jure measures include variables that represent policies that, in principle, facilitate flows and activities” [44]. In our study, we assessed the impact of overall economic globalization on food insecurity. To mitigate possible multicollinearity risks, as both trade and financial globalization are components of the economic globalization index, while economic globalization is in turn a component of the overall globalization index, we have chosen to model only the impact of economic globalization.

Trade globalization has the potential to increase availability, affordability, and access to healthy food and thus reduce food insecurity [47,48]. By facilitating imports of food that happen to be locally short or seasonally out of season, world trade stabilizes supply and cushions against national production shocks such as droughts or pests [49]. It has the potential to decrease price volatility and make food more affordable by increasing competition and diversification of supply. Additionally, trade facilitates access to agricultural inputs, innovations, and technologies that enhance domestic food production. Increased integration in global markets can lead to income and employment in the export sector, enabling households to buy higher-quality and larger quantities of food. Furthermore, access to a wider variety of imported foods can enhance dietary diversity and improve nutrition. However, the benefits from trade are contingent upon domestic economic institutions, infrastructure, and whether gains are equitably distributed to society [50].

Household final consumption expenditure (HFCE) indicates household behavior and purchasing power, functioning as a significant indicator of economic activity. They quantify the expenditure of households on goods and services, encompassing both long-term and short-term consumption, as well as on housing and public services [51,52]. A recent study found that per capita HFCE is the strongest and most consistent predictor of national food security, explaining most of the variation in both the Global Food Security Index and the Food Insecurity Experience Scale. Other factors, such as agricultural land, cereal yields, governance, and employment, show weak or inconsistent effects in the case of this study [53].

The unemployment rate is a significant aspect for a country’s economic and financial growth, and can become a driver of food insecurity [54,55]. The unemployment rate is the share of the “labor force that is without work but available for and seeking employment” [56,57]. The labor force comprises the total number of employees, the self-employed, unpaid family members, and the unemployed. Unemployed individuals are those of working age who lack employment, are available for work, and have been actively looking for employment in the preceding four weeks [58].

The second variable category relates to food and agriculture and is composed of the following variables:

Food price anomalies denote unusually high or low market prices for food goods. The indicator is based on a weighted compound growth rate that considers price increases both within and across years. The indicator assesses monthly price growth over several years, taking into account seasonality in agricultural markets and inflation, to determine whether a price change is atypical for a specific period. The approach is utilized for both individual food commodities and a collection of food products [59,60]. In our case, we assessed the average value of the food price anomaly for years 2021–2023.

The cereal import dependency ratio (*CIDR*) indicates the proportion of the domestic cereal supply that is imported compared to the amount produced domestically [61,62]. *CIDR* is calculated as:

$$CIDR=\;\frac{Ci-Ce}{Cp+Ci-Ce}*100$$

where: *Ci* represents cereal imports; *Ce*, cereal exports; and *Cp*, the nationwide cereal production. According to this formula, the indicator exclusively assumes values less than or equal to 100. Negative values signify that the country is a net exporter of cereals [26].

The economic output of agriculture is quantified in our study through the value of agricultural production per unit area, expressed in dollars per hectare after accounting for purchasing power parity. This variable illustrates the effectiveness and intensity of land use for farming. While lower values suggest less intensive farming, lower yields, or subsistence-level production, higher values usually indicate increased productivity and/or higher-value crops being grown [63,64].

Agricultural land denotes the portion of land that is arable, cultivated with permanent crops, and designated for animal husbandry. Arable land encompasses areas classified by the FAO as land utilized for temporary crops (with double-cropped regions counted singularly), temporary meadows designated for mowing or grazing, land allocated for market or kitchen gardens, and land that is temporarily fallow. Land forsaken due to changing cultivation is excluded. Land designated for permanent crops is utilized for crops that remain in place for extended durations and do not require replanting after each harvest, including cocoa, coffee, and rubber. This category encompasses territory occupied by shrubs, fruit trees, nut trees, and vines, while excluding property designated for trees cultivated for wood or timber production. Permanent pasture refers to land utilized for fodder for five years or longer, encompassing both natural and produced crops [65].

The following variable category consists of governance, political stability, and war, and is composed of:

Political stability and the absence of violence assess perceptions regarding the probability of government destabilization or overthrow through unconstitutional or violent methods, encompassing politically-motivated violence and terrorism [66,67]. This definition indicates that terrorism is a significant threat to political stability and effective governance. Terrorism encompasses activities, actions, and behaviors aimed at imposing radical ideologies on individuals and political institutions or instilling fear in them. Terrorism disrupts individuals’ daily routines and plans, as well as the economic development of affected areas [30].

War and armed conflicts’ impact is operationalized in our study as the number of deaths among combatants and civilians resulting from armed conflict, expressed per 100,000 people. This measure includes fatalities from all conflicts active during the given year, capturing the direct human toll of warfare on a country’s population [68,69]. This variable was averaged over the 2021–2023 period. We transformed this variable into a dummy, where the value of 1 was assigned to the presence of armed conflicts (non-zero deaths), while 0 was assigned to a lack thereof.

Population and demographics are expressed through the following variables:

Urbanization rates represent the proportion of the total population residing in urban areas and are influenced by rural-to-urban migration and natural population growth. Urbanization experienced nearly exponential expansion from the late 19th century. This process is closely associated with the introduction of new technologies, particularly those facilitating food production, improved public health and sanitation, as well as mass mobility, such as the railroad [31]. Urbanization rates are expected to have a positive impact on food security (and an inverse relation with food insecurity), due to the high rates of economic growth and improved access to market infrastructure, however these factors might be trumped by an overt reliance on purchased food which is not as present in rural areas, where food production can be sustained by the household in many cases.

Population refers to the total number of inhabitants residing in a specific area (such as a country or the world), which is continuously altered by increases (births and immigration) and decreases (deaths and emigration). The expansion of a human population is constrained by food availability, prevalence of diseases, and many environmental conditions [70].

Lastly, the impact of natural disasters is assessed by the percentage of GDP lost due to them.

## 3. Materials and Methods

### 3.1. Data Sources and Processing

This study employs a quantitative, cross-sectional analysis that covers the latest data reported on food insecurity and its potential influencing factors, based on a dataset of 153 countries. Primary data sources include the World Bank’s World Development Indicators and other relevant databases, through the WDI R package (version 2.7.9) [42,71], FAO’s Food Security indicators (FAOSTAT) [29,72,73], the KOF Globalization Index [44], the World Inequality Database [74], as well as through the Our World in Data aggregation portal through the owidR package [75]. The data used are publicly available through the institution’s website. In the case of the dependent variable, the unit was scaled to the [0, 1] interval. The data are available as Supplementary Material, in Table S1.

Table 3 shows the units in which the variables are expressed, the ratio of missing values per variable, as well as the statistical procedures employed for data cleanup and imputation.

The overall rate of missingness in the dataset is 2.76%. The variable with the highest amount of missing values is per capita HFCE, with 16.33% of values missing. Those results prompted us to use Multiple Imputation by Chained Equations (MICE), which has been described as “a robust, informative method of dealing with missing data in datasets” [76,77]. According to methodological guidelines in statistical literature, analyses involving variables with more than 10% missing data carry an increased risk of bias. Furthermore, if key variables exceed a missingness threshold of 40%, analytical results should be interpreted cautiously and considered as hypothesis-generating procedures rather than conclusive analyses [78,79]. In the case of our dataset, the ratio of missing variables is sufficiently low to justify both the use of the MICE algorithm and the overall trust in the analysis results.

Some extreme values were detected in the raw dataset. The beta regression is sensitive to outliers and extreme values [80]. We implemented the winsorizing technique on a limited number of variables, as described in Table 2. Descriptive statistics before and after winsorizing are reported in the Results section of the article.

### 3.2. Analytic Method: Beta Regression

Given that the dependent variable is a proportion, we employed a beta regression model to estimate the effects of the predictors on food insecurity using the betareg R package [24]. Beta regression is designed explicitly for continuous outcomes bounded in the unit interval (0,1), as is the case with moderate or severe food insecurity, and keeps fitted values within this range while allowing the variance to depend on the mean, which addresses the strong heteroskedasticity typical of this type of data [24]. In this approach, the response variable is assumed to follow a beta distribution, which is highly flexible in shape and can accommodate skewed, heteroskedastic data much better than the traditional OLS regression models [81].

By contrast, OLS usually assumes normal, homoskedastic errors and can yield out-of-bounds predictions (for example, negative food insecurity, which would have no correspondence to reality) and misleading standard errors.

The trade-offs are that standard beta regression excludes exact 0 s or 1 s, requiring zero/one-inflated or adjusted variants (however, this is not an issue in our study, as the range of the dependent variable does not include 0 or 1) and the fact that it relies on pseudo-R^2^ with somewhat more involved diagnostics; however, these costs are outweighed by the improved fit and more reliable, policy-relevant marginal effects on the mean scale [82,83,84].

The beta density parametrization (1) is expressed as:

(1) $$f\left(y,\;\mu ,\;\phi \right)=\;\frac{\Gamma \left(\phi \right)}{\Gamma \left(\mu \phi \right)\Gamma \left(\left(1-\mu \right)\phi \right)}y^{\mu \phi -1}{\left(1-y\right)}^{\left(1-\mu \right)\phi -1}$$

0<y<1

where:

*Γ* is the gamma function;

ϕ is the precision parameter (ϕ > 0);

*μ* is the mean of the response variable;

The beta regression model for the mean (2) is defined as:

(2) $$g\left(\mu_{i}\right)=\;x_{i}^{T}\beta$$

where:

*g*(∙) is the link function;

$x_{i}^{T}$ is the vector of predictors;

*β* are regression coefficients.

In the beta regression model, the mean of the beta-distributed outcome is linked to the set of independent variables via a link function (logit, probit, cloglog, cauchit, log, loglog). Choosing the appropriate link function is an important part of model specification. We provide a comparison between all but the log link function (which was not used because some independent variables contained negative values, and the logarithm of a negative value is undefined) in Table 4, containing the Akaike Information Criterion (AIC), Bayesian Information Criterion (BIC), and a pseudo R^2^ (based on the squared correlation between transformed response and linear predictors across link functions).

Based on the results of the AIC and BIC in particular (which are stronger indicators than the pseudo R^2^), we chose the Cauchy link, which is based on the inverse of the cumulative distribution function of the Cauchy distribution. It is heavier-tailed than the logit and probit links and can offer greater robustness to outliers in the predictor space or to extreme values in the dependent variable. Direct interpretation of coefficients is less intuitive than would be in the case of the logit link (where the coefficients obtained signify changes in the log-odds of the dependent variable), and marginal effects or predicted values are discussed in the Results section to aid interpretation.

The beta regression model was extended to include a precision submodel, allowing the dispersion of the dependent variable to vary as a function of per capita HFCE. In the betareg framework, this results in a two-part model: one equation models the mean of the response (linked via a cauchit function), while a second models the precision parameter (φ), which controls the variability (inverse dispersion) of the beta distribution.

Including HFCE in the precision submodel enables the model to account for heteroskedasticity, allowing the variability of food insecurity prevalence across countries to depend on their level of economic development or household spending capacity. Specifically, countries with higher HFCE may have not only lower average food insecurity but also more stable and predictable food security conditions, resulting in higher precision (lower variance) around the mean.

This modeling choice enables beta regression to more accurately reflect the data-generating process by relaxing the assumption of constant precision. Model selection criteria (AIC and BIC) indicated that including HFCE in the precision submodel improved model fit without introducing instability (we observed a decrease from −248.73 to −272.77 in the AIC and from −191.15 to −212.17 in the BIC, respectively).

Estimation was carried out by maximum likelihood, as implemented by betareg (which uses analytical gradients and Fisher scoring for stable convergence).

**Figure 1:**
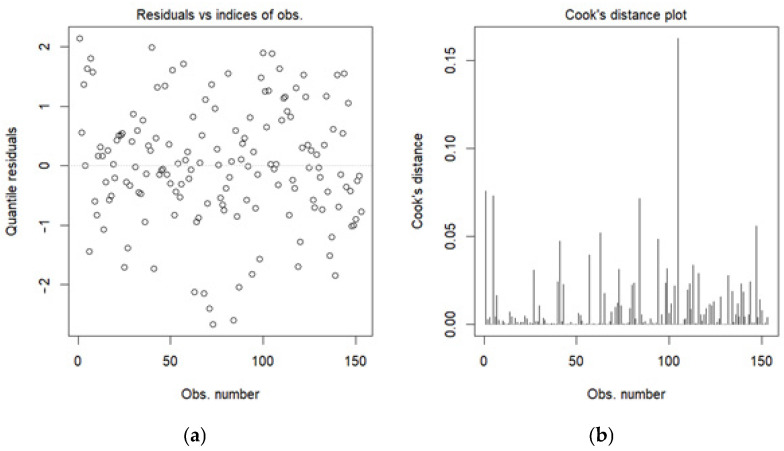
Residuals vs. indices of observations (**a**) and Cook’s distance plots (**b**).

**Figure 2:**
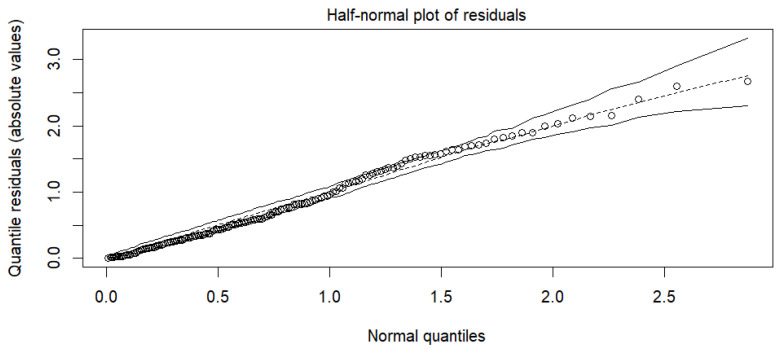
Half-normal plot of residuals.

**Figure 3:**
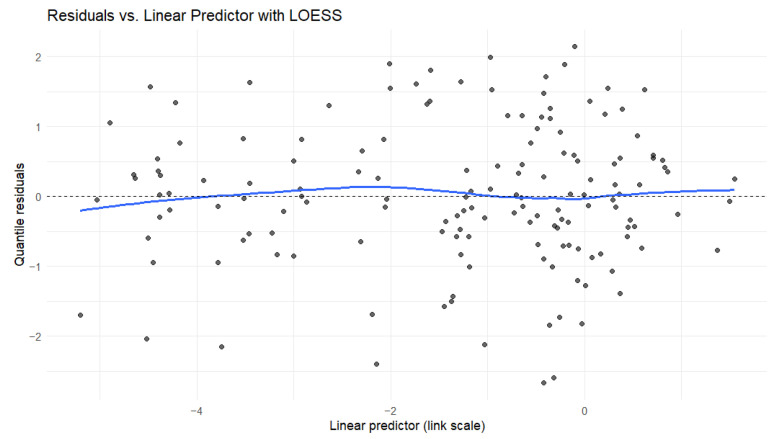
Residuals vs. linear predictor plot with LOESS.

**Figure 4:**
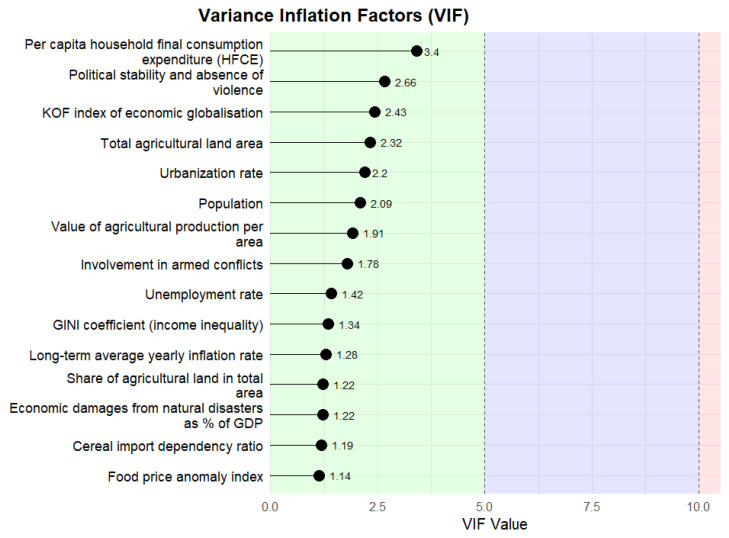
VIF values for independent variables.

Further model diagnostics were performed to ensure the beta regression assumptions were met. We examined residuals vs. indices of observations for link appropriateness (Figure 1a), the Cook’s distance for lack of outliers (Figure 1b), the residuals vs. linear predictor plot for linearity and homoskedasticity (Figure 2), the half-normal plot of deviance residuals for normality (Figure 3), as well as the VIF values for multicollinearity (Figure 4). The residuals vs. indices of observation were uniformly dispersed. The Cook’s distance plot shows a lack of outliers in the model (considering a threshold of 1). Model normality is shown in the half-normal plot of residuals. The residuals did not exhibit patterns indicating misspecification, and no strong multicollinearity was detected among the predictors (variance inflation factors showed a maximum value of 3.4, far below the prescribed threshold of 5 or 10).

All analyses and plotting were done in R (version 4.4.3) with the tidyverse (version 2.0.0), betareg (version 3.2-3), car (version 3.1-3) and performance (version 0.14.0) packages [24,85,86,87,88].

## 4. Results

### 4.1. Descriptive Statistics

The descriptive statistics for the variables used in the beta regression model are shown in Table 5 (n = 153). The proportion of the population experiencing moderate or severe food insecurity ranges from 2% to 89%, with an average of 33%, a standard deviation of 0.25 (25%), a moderately low skew of 0.63, and a kurtosis of −0.81, indicating a relatively symmetric and slightly platikurtic distribution in the dependent variable.

The GINI coefficient (mean = 0.50, SD = 0.11) and long-term average annual inflation rate (mean = 6.50%, SD = 9.20%) display varying degrees of dispersion and asymmetry, with the latter exhibiting positive skew (skew = 3.61) and high kurtosis (14.12), indicating some slight upper-end extreme values and a leptokurtik distribution. Per capita HFCE also exhibits a long right tail (mean ≈ 11,619 PPP$, max ≈ 34,904 PPP$), suggesting the presence of some extreme values. However, the influence of these values is limited, as shown in the Cook’s distance plot, and does not affect the regression.

Variables such as agricultural production per area and total agricultural land area show large ranges and high dispersion, with minimum values close to zero and maximum values exceeding 10,000 and 112,000, respectively. The KOF index of economic globalization has a relatively concentrated distribution (mean = 0.58, SD = 0.17), while unemployment rates (mean = 8%) and urbanization (mean = 60%) vary more moderately. The political stability indicator, scaled to a 0–100 range, has a mean of 50 but spans a wide range (−0.10 to 81.54 after rescaling), and involvement in armed conflict, measured as a binary variable, was recorded in 25% of countries.

### 4.2. Beta Regression Model Results and Marginal Effects of Important Variables

The results presented in Table 6 summarize the beta regression analysis conducted using the Cauchy link function for the mean model and a log link for the precision model, as described in the Materials and Methods section, examining the determinants of national-level food insecurity worldwide.

In the mean model, several independent variables were observed as being statistically significant. The intercept (−1.416, *p* = 0.02) indicates a low baseline level of food insecurity when all other explanatory variables are set to zero. However, it should be noted that most of the independent variables in the model, most notably per capita HFCE, population, agricultural production, and land, among others, cannot realistically take the value zero. As such, the negative sign of the intercept is not unexpected, but is in itself meaningless for statistical interpretation.

The GINI coefficient displayed a strongly significant positive association (0.047, *p* < 0.001), indicating the adverse impact of income inequality on food security and confirming previous studies [89,90,91]. Similarly, the average yearly inflation rate (0.018, *p* = 0.003) was positively correlated with food insecurity, suggesting that economic instability and consistently high inflation rates exacerbate food security issues [92].

Conversely, higher economic globalization, represented by the KOF Globalization Index (−0.021, *p* = 0.001), showed a significant negative effect, implying that openness to international trade and finance may mitigate food insecurity through improved resource allocation (by allowing country-level economic specialization and taking advantage of comparative advantage) and access to food commodities. The per capita HFCE also exhibited a highly significant negative relationship (−0.00009, *p* < 0.001), indicating that greater household consumption capacity substantially enhances national food security. This is consistent with previous research, showing that HFCE is among the most important factors influencing food insecurity.

Other variables showed mixed or marginal results. The total agricultural land area (−0.00001, *p* = 0.02) was negatively associated with food insecurity, albeit with a small effect size, reflecting the protective role of extensive agricultural resources. Agricultural productivity per hectare showed marginal statistical significance (−0.00009, *p* = 0.09), suggesting potential benefits from improved agricultural efficiency, although the evidence remains weak.

Contrary to expectations, several predictors were not statistically significant. Unemployment rate (−0.008, *p* = 0.41), food price anomalies (−0.003, *p* = 0.98), cereal import dependency (0.001, *p* = 0.43), the percentage of agricultural land (0.002, *p* = 0.52), political stability (0.001, *p* = 0.87), urbanization rate (0.003, *p* = 0.44), population size (−0.001, *p* = 0.42), disaster-related GDP damage (0.070, *p* = 0.36), and the presence of conflict (war dummy, 0.044, *p* = 0.77) did not significantly explain the variation in food insecurity in the analyzed sample. These findings suggest that economic variables are the most important ones in predicting food insecurity.

The precision model revealed that per capita HFCE has a positive influence on the precision of the estimates (7.23 × 10^−5^, *p* < 0.001), indicating greater consistency and lower variability in food insecurity levels within countries with higher consumption expenditures. The intercept in the precision model (1.996, *p* < 0.001) confirms substantial baseline precision.

To interpret the beta regression coefficients more intuitively, the marginal effects of statistically significant independent variables on food insecurity were calculated and plotted. The marginal effects plots show the effect of a specific independent variable, while holding all others constant at their average values.

**Figure 5:**
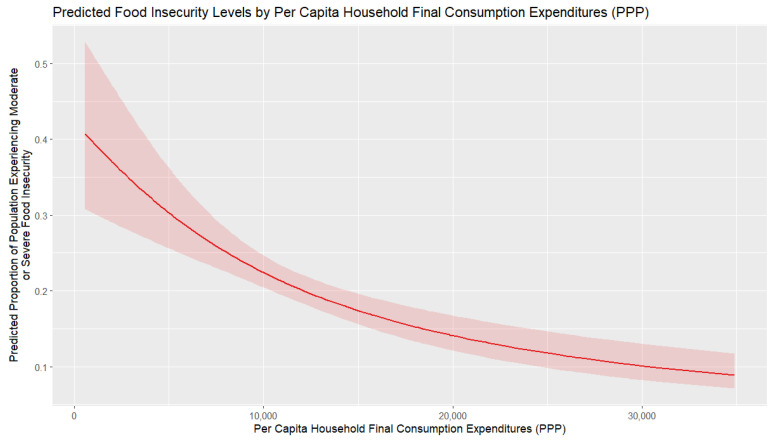
Marginal effect of per capita HFCE on food insecurity.

Figure 5 illustrates the marginal effect of per capita HFCE on food insecurity, with all other variables in the regression model held at their mean values. A noticeable result is that higher HFCE reduces food insecurity, but the impact is strongest at low-income levels and weakens as consumption levels increase. Marginal effects diminish with higher expenditures, indicating that boosting consumption in poorer populations is most effective for reducing food insecurity. In contrast, for countries with higher levels of consumption, the associated decrease in food insecurity tends to reach a plateau, all other things being equal. It is worth noting that additional calculations reveal a strong correlation between per capita HFCE and per capita GDP (with a correlation coefficient of 0.91); thus, overall economic development can be considered a significant driver of food security.

**Figure 6:**
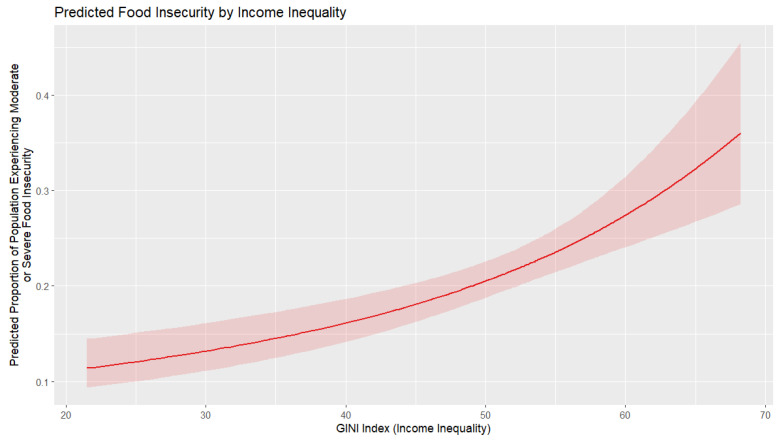
Marginal effect of income inequality on food insecurity.

Figure 6 shows that higher income inequality (GINI index) increases food insecurity, with the marginal effect also growing stronger as inequality rises. The impact is modest at low income inequality levels but accelerates as the GINI index rises. As such, reducing inequality is thus key to mitigating food insecurity, especially in highly unequal countries. Higher inequality reduces the impact of economic growth on personal well-being and, by extension, on food security.

**Figure 7:**
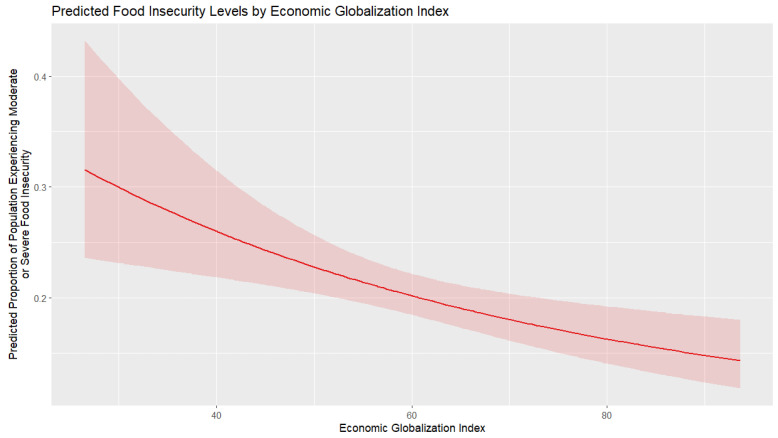
Marginal effect of economic globalization on food insecurity.

Figure 7 shows the marginal effects of economic globalization on moderate or severe food insecurity. A clear inverse relationship is observed, indicating that food insecurity levels tend to decrease as economic globalization increases. This influence is at its maximum in countries with very low levels of globalization, where even marginal changes can lead to big drops in food insecurity. Marginal benefits decrease as countries become more globalized; therefore, the additional steps of global economic integration bring smaller increments to food security. It is also observed that the confidence bands become wider at lower globalization levels; therefore, the impact of economic globalization shows more uncertainty in those intervals, even though the overall trend is clear.

**Figure 8:**
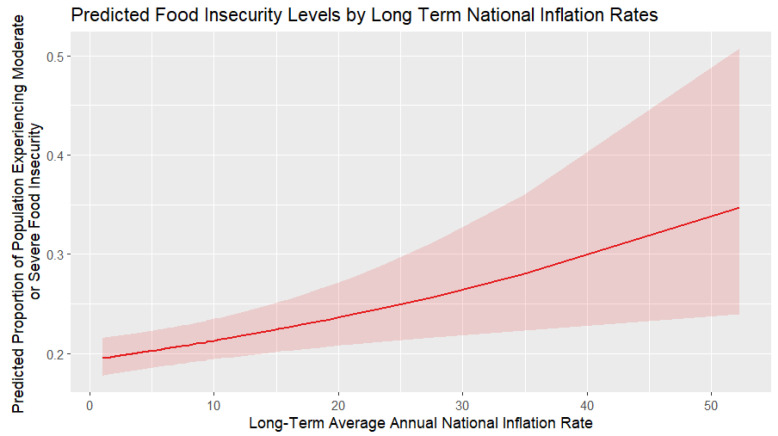
Marginal effect of long-term average inflation rates on food insecurity.

Figure 8 shows the marginal effects of long-term average annual national inflation rates on the ratio of population experiencing moderate or severe food insecurity. The marginal effect is consistently positive, indicating that higher inflation rates increase the likelihood of food insecurity. The effect strengthens as inflation rises, as the impact of inflation becomes more severe at higher inflation rates, particularly beyond 20–30%. However, the widening confidence intervals at elevated inflation levels suggest more uncertainty in these extreme cases. Overall, this suggests that inflation has a significant impact on food insecurity, making price stability a crucial factor in ensuring food security. Short-term food price shocks in the studied interval are not a statistically significant factor in predicting food insecurity, while long-term inflation is. This is of particular interest to policymakers, as they should create regulations and strategies that ensure long-term price stability.

**Figure 9:**
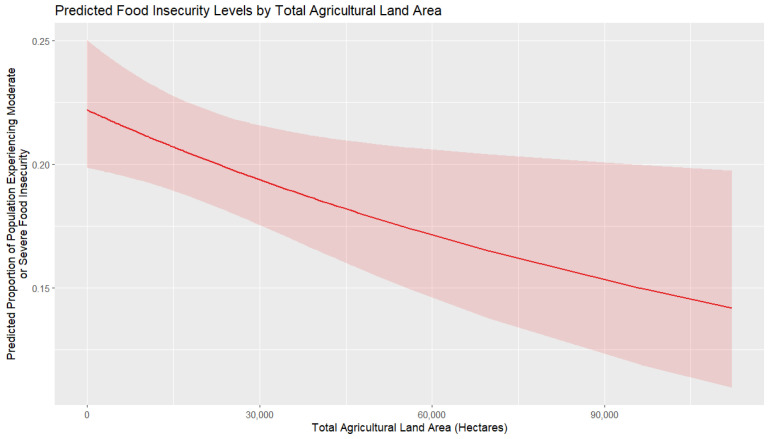
Marginal effects of agricultural land area on food insecurity.

Figure 9 shows the marginal effects of total agricultural land area in the studied countries on the predicted percentage of population experiencing moderate or severe food insecurity. Overall, the marginal effect is consistently negative, indicating that as the total agricultural land area increases, food insecurity decreases. The decline is gradual, suggesting that the expansion of agricultural land contributes steadily to reducing food insecurity. The confidence interval narrows slightly in countries with low agricultural land area, implying that other, more significant factors might be at play in those areas, perhaps economic factors. Overall, the results suggest that larger agricultural land resources are associated with lower levels of food insecurity; however, the certainty of the results tends to decrease with increasing land areas. From a practical point of view, increasing agricultural land areas may not be a feasible way to decrease food insecurity, except in cases where desertification and other factors reducing usable agricultural land are at play.

**Figure 10:**
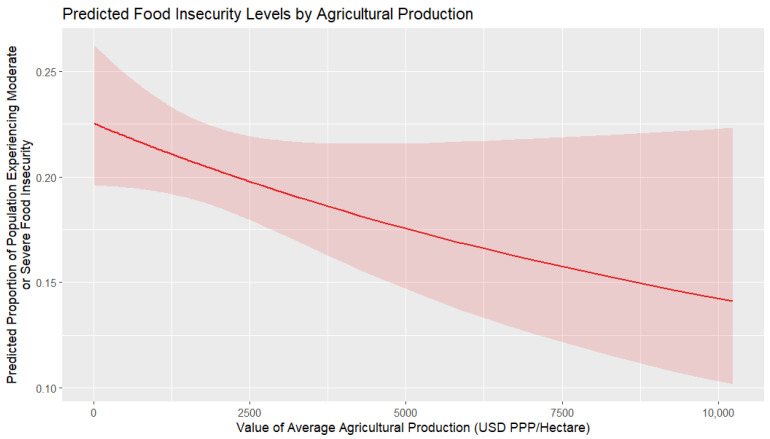
Marginal effects of average agricultural production per hectare on food insecurity.

Figure 10 shows the marginal effect of agricultural production per hectare on food insecurity. The relationship between the two variables is negative, meaning that as the value of the agricultural output per hectare increases, the ratio of food insecurity decreases. There is a more substantial reduction in food insecurity at lower levels of production value, while the rate of decrease slows at higher values. The confidence interval is wider at the extremes, particularly at very low and very high production values, indicating greater uncertainty in these ranges due to the relatively small number of countries with extremely high or low agricultural production. This suggests that increasing agricultural productivity per hectare is associated with lower food insecurity, particularly in contexts where the initial agricultural value is low.

In plain terms, our results say that economic development, spending, access to global markets, and equality influence food insecurity far more than farm capacity and agri-food production do. Countries where households can spend more (higher HFCE) have markedly lower food insecurity, with the effect more pronounced especially at low income levels, where each extra unit of spending goes furthest—while the benefit tapers at higher incomes. Where income is unequally distributed (higher Gini), more people struggle to afford adequate diets, even if the economy is growing. Persistent inflation also worsens food insecurity by steadily eroding purchasing power. By contrast, being more integrated into the global economy (economic globalization) is associated with lower food insecurity, likely because diversified trade helps keep markets supplied and prices in check. Agricultural resources and productivity (more land, higher value per hectare) help, but their effects are modest compared with these economic forces. Several variables—short-run food price anomalies, cereal import dependency, unemployment, urbanization, population, political stability/violence, disaster damages, and conflict—are not statistically significant once we account for purchasing power, inflation, and global openness. Finally, HFCE also raises the model’s precision, meaning food insecurity is not only lower but also more predictable when households are better able to spend. In terms of testing the hypotheses formulated, we fail to reject the null for all but H1, H2, H3, H4, H8, and H9.

## 5. Discussion

The key findings of this research indicate that the most important factors influencing food insecurity are related to final household consumption, income inequality, openness to international trade and globalization, and inflation. In contrast, agricultural production and land areas of the studied countries represent statistically significant, but weaker, factors. This is an indicator of the fact that economic factors primarily drive global food insecurity crisis.

In this regard, policy recommendations aimed at fulfilling SDG goals should focus on helping low-income countries achieve sustainable development, reducing income (and, by extension, social) inequities, mitigating long-term inflation, and promoting fair and mutually beneficial trade. At the same time, the worldwide dissemination of novel agricultural technologies is also a means to decrease food insecurity; however, policy should focus on addressing the root economic causes. Despite recent setbacks in globalization, the classical liberal theory of comparative advantages suggests that export-oriented agriculture systems in countries specializing in this field are key to reducing food insecurity worldwide. Inefficiencies in food distribution and weak linkages between production value and access to food are considered by us to be important factors affecting food security. As such, public investment should be prioritized where marginal returns are highest—jurisdictions with low household consumption capacity and weak market integration—and, where baseline agricultural value per hectare is low, directed to productivity-enhancing, climate-smart technologies, and rural infrastructure. Beyond these implications, our estimates point to several refinements that future research and policy could operationalize, such as adopting inequality-sensitive hunger metrics (e.g., FIES disaggregated by income quintile or a Gini-weighted FIES) to ensure economic growth translates into tangible improvements in human development; tracking trade-resilience indicators such as import-source diversification for core staples; and quantifying social-protection effectiveness metrics (for example, the coverage of school feeding programs and explicit inflation indexation of lower incomes) to protect purchasing power.

Furthermore, the marginal effects plots show diminishing returns for almost all variables of interest. This is important, as it shows that policy recommendations should focus specifically on developing countries to foster economic development.

The regression analysis reveals that some variables considered theoretically important are not statistically significant. Interestingly, cereal import dependency is not a significant driver of food insecurity. This is further evidence that a robust trade system is the most effective way to ensure equitable access to food, as reliance on imports does not negatively impact food security levels. It should be noted that a possible cause for this behavior in the regression analysis is the aggregation at the country level, which might mask local effects.

Our results are consistent with cross-national evidence that economic indicators such as HFCE are among the strongest correlates of food security or its lack thereof [53] and with research emphasizing the roles of inequality and social protection in shaping access to food [89,90,91]. The positive association between persistent inflation and food insecurity aligns with macro evidence that price increases push households into poverty and undermine dietary quality [14,92]. The negative link between economic globalization and food insecurity echoes arguments and network evidence that trade improves availability and buffers domestic shocks [47,48,49,50].

By contrast, cereal import dependency is not significant in our model once the broader macro and trade context is controlled. This differs from settings where import reliance heightened vulnerability during geopolitical disruptions [61]. Our cross-sectional approach may attenuate such episode-specific effects, and this can be considered a limitation of our study design.

This study has several limitations that should be acknowledged. First, the cross-sectional design restricted the analysis to a single point in time, preventing the assessment of stronger causal relationships or temporal dynamics in food insecurity determinants (where Granger causality or other time series analysis methods might apply). As a result, observed associations may vary across different periods or economic contexts. Second, and related to the first point, methodological constraints also apply: no widely adopted statistical packages currently allow for panel data modeling with beta regression, limiting the ability to exploit longitudinal datasets. Addressing this gap represents a promising direction for future methodological development, with potential benefits for food security research. Third, while our model captures national-level patterns, it does not account for potential spatial heterogeneity. Future research could integrate geographically weighted beta regression to identify and quantify how the effects of explanatory variables differ across regions, thereby offering more targeted policy insights. Finally, as with many large-scale cross-country analyses, the results may be influenced by differences in data quality, measurement standards, and the availability of reliable statistics across countries. Variable selection, though guided by theory and prior research, is also constrained by data availability, which may lead to omitted variable bias and an incomplete representation of the drivers of food insecurity.

The strengths of our study are the robust modelling method, as the beta regression is designed for ratio dependent variables. Additionally, the wide range of independent variables is another strength of our study, which reduces the risk of missing variable bias.

## 6. Conclusions

This study employed a beta regression model to analyze the country-level drivers of moderate or severe food insecurity among 153 countries. The results show that economic factors—specifically, household final consumption capacity, income inequality, inflation, and economic globalization—are the strongest and most consistent predictors of food insecurity, whereas the effects of agricultural factors, such as land and hectare yield, were somewhat modest. This suggests that, at least at the global level, the capacity to pay for food plays a larger role than agricultural supply itself. These findings show the need to promote policies that foster equitable economic growth, turn around inequality, promote price stability, and foster fair and open trade. These policies are likely to produce more substantial and sustainable improvements in food security, particularly in less developed and countries with high income inequality.

Building on the present findings, future research should integrate longitudinal datasets and explore beta regression models adapted for panel data, enabling the assessment of dynamic trends and causal relationships over time. Incorporating geographically weighted beta regression could also uncover spatial variations in the influence of key determinants, guiding more targeted interventions. Furthermore, expanding the analysis to possibly include environmental variables (e.g., climate change impacts, water availability) and other governance indicators could provide a more nuanced understanding of food insecurity drivers. Such methodological and thematic extensions would strengthen the evidence base for designing effective, context-specific policies to achieve the Sustainable Development Goal of Zero Hunger.

In conclusion, in order to successfully fight food insecurity, there must be an integrated strategy of bringing together economic, social, and agricultural policies that are attuned to the specific needs of different countries, while taking into consideration the need to develop emerging countries.

## Figures and Tables

**Table 1:** Evolution of moderate or severe food insecurity aggregated at the regional level (%).

	Year	2015	2016	2017	2018	2019	2020	2021	2022
Region									
Northern Africa		28.6	29.6	31.2	30.8	30	31	32.2	33.4
Eastern Africa		58.5	61.1	63	62.9	63.3	64.2	65.6	65.4
Middle Africa		N/A	N/A	N/A	N/A	69.5	71.7	74.1	76.7
Southern Africa		21.5	21.7	21.8	21.9	22.8	23.7	24	24.1
Western Africa		39.7	42.5	45	46.9	50	54.6	58.3	60.7
Latin America and the Caraibbean		25.1	27.5	28.7	28.9	30.5	32.6	33.4	31.3
Central America		28.9	28.1	27.6	28.4	30.5	31.7	31.3	29.3
South America		19.7	23.7	25.7	25.9	27.3	30	31.4	29.2
Northern America		9.9	9.3	8.5	8.1	8	7.8	8.5	9
Central Asia		9.2	11	12.5	13.7	15	17.2	18.4	18
Eastern Asia		6	7.4	8.6	9	8.2	7.1	6.7	6.2
South-eastern Asia		14.8	15.2	15.3	15.1	15.1	15.7	16.5	17
Southern Asia		27.6	27	28.4	30.7	36.4	39.8	42	41.3
Western Asia		30.7	31.6	31.7	32.3	34	37	38.9	38.9
Eastern Europe		11.2	11.2	10.3	9.2	9.2	9.7	10.4	10.6
Northern Europe		6.7	6.5	6	5.5	4.9	4.6	5.1	6.3
Southern Europe		7.4	7.6	7.4	7.5	7.3	7.3	7.1	6.5
Western Europe		5.2	4.9	4.7	4.5	4.2	4.4	4.9	5.6
Oceania		22.2	23	23.8	24.3	23.8	23.8	23.8	25
World		21.7	22.5	23.6	24.5	26.2	27.7	29	29

Source: [29].

**Table 2:** Possible causes of food insecurity.

Variable Type	Variable Name	Expected Relation	Hypothesis No.
Economic	GINI coefficient (income inequality)	Positive	H1
	Long-term average yearly inflation rate	Positive	H2
	KOF index of economic globalization (trade and financial openness to global markets)	Negative	H3
	Per capita household final consumption expenditure (HFCE)	Negative	H4
	Unemployment rate	Positive	H5
Food and agriculture	Food price anomalies index	Positive	H6
	Cereal import dependency ratio	Positive	H7
	Value of agricultural production per area	Negative	H7
	Total agricultural land area	Negative	H8
	Share of agricultural land in total area	Negative	H9
Governance, political stability, and war	Political stability and absence of violence	Negative	H10
	Death rate in armed conflicts	Positive	H11
Population and demographics	Population	Positive	H12
	Urbanization rate	Negative/Positive	H13
Disasters	Economic damages from natural disasters as % of GDP	Positive	H14

**Table 3:** Variable units and data processing techniques applied.

Variable Name	Unit	Missing Values Percentage	Data Procedure
Proportion of population experiencing moderate or severe food insecurity	Percentage	0%	None
GINI coefficient (income inequality)	Unitless	0%	None
Long-term average yearly inflation rate	Percentage	0.65%	MICE
KOF index of economic globalization (trade and financial openness to global markets)	Unitless	3.26%	MICE imputation
Per capita HFCE	US Dollars PPP (2021)	16.33%	MICE, winsorizing (99% quantile)
Unemployment rate	Percentage	3.92%	MICE
Food price anomalies index	Unitless	1.30%	MICE
Cereal import dependency ratio	Percentage	6.53%	MICE
Value of agricultural production per area	Dollars/Hectare	0%	Winsorizing (1–99% quantiles)
Total agricultural land area	1000 Hectares	0%	Winsorizing (5–95% quantiles)
Share of agricultural land in total area	Percentage	0%	None
Political stability and absence of violence	Unitless	0%	None
Involvement in armed conflicts	Dummy variable of involvement in armed conflicts	0%	None
Population	Millions	0%	None
Urbanization rate	Percentage of population	4.57%	MICE
Economic damages from natural disasters as % of GDP	Percentage of GDP	0%	Winsorizing (1–99% quantiles)

**Table 4:** Goodness of fit statistics for the beta regression model.

	Logit	Probit	Cloglog	Cauchit	Loglog
AIC	−246.12	−237.40	−254.08	−270.58	−221.57
BIC	−191.57	−182.85	−199.53	−216.03	−167.02
Pseudo R^2^	0.72	0.72	0.72	0.70	0.71

**Table 5:** Descriptive statistics.

Variable Name	Mean	Standard Deviation	Median	Median Absolute Deviation	Minimum	Maximum	Range	Skew	Kurtosis
MFSI	0.33	0.25	0.28	0.28	0.02	0.89	0.87	0.63	−0.81
GINI coefficient	0.50	0.11	0.53	0.09	0.22	0.68	0.47	−0.84	−0.24
Long-term average yearly inflation rate	6.50	9.20	3.60	2.27	1.05	52.30	51.25	3.61	14.12
KOF index of economic globalization (trade and financial openness to global markets)	0.58	0.17	0.58	0.20	0.26	0.94	0.67	0.04	−0.99
Per capita HFCE	11,619.01	9293.02	8812.74	9116.14	580.03	34,903.91	34,323.88	0.76	−0.58
Unemployment rate	0.08	0.06	0.06	0.04	0.00	0.37	0.37	1.85	4.37
Food price anomalies index	0.53	0.56	0.51	0.58	−0.64	2.09	2.73	0.37	−0.43
Cereal import dependency ratio	0.27	0.84	0.43	0.59	−4.76	1.00	5.76	−2.76	10.86
Value of agricultural production per area	1749.70	2005.99	1100.03	1102.56	16.91	10,230.30	10,213.39	2.39	6.17
Total agricultural land area	16,836.58	27,537.35	3941.03	5780.70	8.75	112,209.94	112,201.19	2.29	4.79
Share of agricultural land in total area	0.39	0.21	0.41	0.23	0.00	0.84	0.84	0.10	−0.80
Political stability and absence of violence	50.00	20.00	51.04	21.23	−0.10	81.54	81.64	−0.69	−0.28
Urbanization rate	0.60	0.22	0.64	0.26	0.11	1.00	0.89	−0.24	−0.94
Population	30.10	53.83	9.69	13.11	0.05	341.72	341.68	3.20	11.66
Economic damages from natural disasters as % of GDP	0.00	0.01	0.00	0.00	0.00	0.05	0.05	5.81	33.49
Involvement in armed conflicts	0.25	0.43	0.00	0.00	0.00	1.00	1.00	1.15	−0.67

**Table 6:** Coefficients for the mean and precision models.

Variable Name	Estimate	Std. Error	z Value	Pr (>|z|)
Coefficients (mean model with cauchit link)				
(Intercept)	−1.41586	0.63	−2.26	0.02 *
GINI coefficient	0.04716	0.01	4.80	0.00 ***
Long-term average yearly inflation rate	0.01761	0.01	2.97	0.00 **
KOF index of economic globalization (trade and financial openness to global markets)	−0.02112	0.01	−3.18	0.00 **
Per capita HFCE	−0.00009	0.00	−5.00	0.00 ***
Unemployment rate	−0.00804	0.01	−0.83	0.41
Food price anomalies index	−0.00250	0.10	−0.02	0.98
Cereal import dependency ratio	0.00066	0.00	0.79	0.43
Value of agricultural production per area	−0.00009	0.00	−1.69	0.09
Total agricultural land area	−0.00001	0.00	−2.25	0.02 *
Share of agricultural land in total area	0.00180	0.00	0.64	0.52
Political stability and absence of violence	0.00071	0.00	0.16	0.87
Urbanization rate	0.00298	0.00	0.78	0.44
Population	−0.00127	0.00	−0.80	0.42
Economic damages from natural disasters as % of GDP	0.07014	0.08	0.91	0.36
Involvement in armed conflicts	0.04448	0.16	0.29	0.77
Phi coefficients (precision model with log link)				
(Intercept)	1.99563	0.17	11.42	0.00 ***
Per capita HFCE	7.23 × 10^−5^	1.2 × 10^−5^	6.026	0.00 ***

The asterisks in the Pr column represent *p* values reported in the regression model: “***” corresponds to a *p* value of ≤0.001, “**” to ≤0.01, “*” to ≤0.05. No asterisk denote a lack of statistical significance.

## Data Availability

The original contributions presented in the study are included in the Supplementary Material. Further inquiries can be directed to the corresponding author/s.

## Supplementary Materials

The following supporting information can be downloaded at: https://www.mdpi.com/article/10.3390/foods14172997/s1, Table S1: Dataset used in study.
